# From Contusions to Fatal Atlanto-Occipital Dislocations: The Clinical Spectrum of Pediatric Horse-Related Trauma over 22 Years

**DOI:** 10.3390/pediatric18050126

**Published:** 2026-09-25

**Authors:** Britta Chocholka, Lara Marie Bogensperger, Vanessa Groß, Antonia Schwarz, Thomas Tiefenboeck, Stephan Payr, Manuela Jaindl

**Affiliations:** 1 Department of Trauma Surgery, University Clinic of Orthopedics and Trauma Surgery, Medical University of Vienna, Waehringer Guertel 18-20, 1090 Vienna, Austria; lara.bogensperger@meduniwien.ac.at (L.M.B.); gross.vanessa@gmx.at (V.G.); n12220006@students.meduniwien.ac.at (A.S.); thomas.tiefenboeck@meduniwien.ac.at (T.T.); stephan.payr@meduniwien.ac.at (S.P.); manuela.jaindl@meduniwien.ac.at (M.J.); 2 Section of Pediatric Trauma Surgery, Department of Trauma Surgery, University Clinic of Orthopedics and Trauma Surgery, Medical University of Vienna, 1090 Vienna, Austria

**Keywords:** pediatric trauma, horse-related injuries, equestrian injuries, injury mechanisms, atlanto-occipital dislocation, craniocervical junction

## Abstract

**Background**: Pediatric horse-related trauma ranges from minor soft-tissue injuries to potentially fatal trauma. Existing studies often focus on hospitalized patients or selected injury patterns, while broader clinical data remain limited. This study aimed to characterize injury mechanisms, patterns, treatment requirements, and outcomes. **Methods**: This retrospective cohort study included patients younger than 19 years treated at a Level I trauma center between 2002 and 2023. Demographics, injury mechanisms, injury patterns, treatment, hospitalization, and outcomes were analyzed. **Results**: A total of 593 patients with 650 injury events were included (mean age at first presentation, 13.0 ± 3.8 years; 90.9% female). Falls predominated (69.8%), followed by kicks (15.2%) and bites (4.6%). Contusions were most frequent (38.5%), followed by fractures (24.5%) and head injuries (16.6%). Injury mechanism was significantly associated with injury pattern (*p* < 0.001; Cramér’s V = 0.43), and age differed across major injury categories (*p* < 0.001). Hospital admission was required in 17.4% of events and operative treatment in 5.2%. Fractures (aOR, 5.43; *p* < 0.001) and head injuries (aOR, 8.85; *p* < 0.001) were independently associated with hospital admission. Follow-up beyond the initial presentation was documented in 236 of 650 events (36.3%). Among the 234 non-fatal events with documented follow-up, 83.3% were free of complaints; these outcome data should be interpreted as conditional on follow-up availability and subject to potential attrition bias. Two of 593 patients (0.3%) died following atlanto-occipital dislocations after falls involving stirrup entrapment and dragging. **Conclusions**: Pediatric horse-related trauma comprises distinct mechanism-specific injury patterns. Although most injuries were managed without hospitalization or surgery, fractures and head injuries accounted for a disproportionate treatment burden, while rare complex mechanisms resulted in fatal craniocervical trauma.

## 1. Introduction

Horse riding and horse-related activities are well-established recreational and sporting pursuits worldwide and are particularly popular across Europe [1,2]. Children and adolescents participate in organized training and competition as well as recreational riding and the routine handling of horses [3]. Despite these benefits, horse-related activities carry a distinct risk of trauma because it combines height and speed with close interaction with a large and potentially unpredictable animal [3,4].

Pediatric horse-related injuries encompass a broad clinical spectrum, ranging from contusions and superficial wounds to fractures, traumatic brain injuries, spinal trauma, visceral injuries and death [3,5]. Previous pediatric studies have reported a marked predominance of female patients, particularly among adolescents, and have identified falls from the horse as the leading mechanism of injury [6,7]. The extremities and head are among the most commonly affected anatomical regions, although injury patterns vary according to the mechanism of trauma and the population studied [8,9]. Fractures are a frequent reason for hospital admission, whereas head injuries account for a substantial proportion of severe and fatal outcomes in selected pediatric trauma cohorts [5,8].

Importantly, horse-related trauma is not confined to falls while riding. Children may also be kicked, bitten, trampled, or otherwise injured during routine contact with a horse [3,10]. Falls while riding are commonly associated with upper-extremity, head, and spinal injuries, whereas direct horse contact in unmounted children is more frequently associated with facial and abdominal trauma [6,9]. These mechanism-specific differences are clinically relevant, because injuries sustained while handling or being in close proximity to a horse may be severe despite occurring from ground level. In a large pediatric trauma-center cohort, unmounted children were younger, sustained more severe injuries, and more frequently required intensive or operative treatment than mounted children [9].

Although fatal outcomes are uncommon, pediatric horse-related trauma may result in severe cranial, spinal, or multisystem injuries [8]. Traumatic atlanto-occipital dislocation represents a rare and highly unstable injury of the craniocervical junction and is predominantly described after high-energy trauma, particularly motor-vehicle collisions. Its clinical consequences may include severe neurological injury, cardiorespiratory arrest, and death [11,12]. Atlanto-occipital dislocation does not appear to have been previously described as a distinct injury entity in larger published pediatric horse-related trauma cohorts [9,13].

Existing pediatric studies are heterogeneous and are frequently derived from hospitalized or trauma-registry populations or focus on selected injury patterns, while the broader clinical spectrum of horse-related trauma and the relationship between specific injury mechanisms, resulting injury patterns, and treatment requirements remain less well characterized [9,13]. The present study therefore aimed to characterize the demographics, mechanisms, anatomical patterns, treatment requirements, and documented outcomes of pediatric horse-related injuries treated at a Level I trauma center over a 22-year period. Two fatal atlanto-occipital injuries following falls from horses are additionally presented as the most severe outcomes.

## 2. Materials and Methods

### 2.1. Ethics, Study Design, and Setting

The study protocol was approved by the Ethics Committee of the Medical University of Vienna on 15 May 2024 (EK 2084/2023). This retrospective single-center cohort study included patients younger than 19 years treated for horse-related injuries at a Level I trauma center between January 2002 and November 2023. Patients initially treated elsewhere were included if subsequent treatment or follow-up was provided at the study center.

### 2.2. Study Population and Data Collection

Cases were identified by searching the institutional database for the German keywords “Reitunfall” for horse-back riding accident and “Pferd” for horse and subsequently verified by medical-record review. Of 753 potentially eligible records, 103 were excluded because they did not represent an injury event involving a real horse (*n* = 40), constituted duplicate records (*n* = 53), or corresponded to erroneous or inaccessible medical records (*n* = 10). The final cohort comprised 650 horse-related injury events in 593 patients. The individual injury event served as the unit of analysis, allowing patients with separate injuries to contribute more than one event.

Extracted variables included age, sex, injury mechanism, protective equipment, diagnosis and anatomical location, imaging, hospital admission, treatment, surgical procedures, length of stay, follow-up, and documented outcome. Mechanisms were categorized as falls, kicks, bites, or other horse-related mechanisms which comprised injuries occurring during riding without a fall, mounting or dismounting, handling or leading a horse, entrapment or compression, collisions, and other mechanisms not attributable to a fall from a horse, kick, or bite (Supplementary Table S1).

Injury patterns were classified as contusions, fractures, head injuries, joint or ligamentous injuries, wounds, visceral injuries, severe craniocervical or spinal injuries, or unclassified injuries. Head injuries included head or craniofacial contusions, concussions, and intracranial hemorrhages; only concussions and intracranial hemorrhages were classified as traumatic brain injuries. Contusions involving other anatomical regions were assigned to the contusion category. Injury classification and assignment of the primary injury category were initially performed by one investigator and subsequently reviewed by the first author. No comprehensive predefined hierarchical ranking of injury categories was applied. In events involving multiple injuries, the clinically most relevant injury was designated as the primary injury category. When a nonspecific soft-tissue diagnosis such as a contusion co-occurred with a more specific structural injury, such as a fracture, the structural injury was classified as the primary injury.

### 2.3. Outcomes and Fatal Atlanto-Occipital Dislocations

Clinical outcomes included hospital admission, operative treatment, persistent symptoms or functional impairment at the final documented assessment, and death. Hospital admission was defined as documented inpatient treatment or a hospital stay of at least one day. Events without documented follow-up were excluded from the outcome assessment and were not classified as either uncomplicated recovery or persistent symptoms. To assess the potential impact of incomplete follow-up, an additional scenario analysis was performed using alternative assumptions for non-fatal events without documented outcome assessment.

The medical records and available imaging of the two fatal atlanto-occipital dislocations were additionally reviewed for mechanism, neurological status, imaging findings, associated injuries, treatment, and clinical course.

### 2.4. Statistical Analysis

Continuous variables are presented as mean ± standard deviation or median with interquartile range, and categorical variables as frequencies and percentages. Associations between injury mechanism and primary injury category were assessed using Pearson’s chi-square test with Monte Carlo estimation for sparse cell counts; Cramér’s V was calculated as effect size. Age differences among the five major injury categories were assessed using the Kruskal–Wallis test followed by Holm-adjusted pairwise comparisons. For additional age-stratified analyses, injury events were grouped according to age at the time of injury as <6 years, 6 to <13 years, 13 to <16 years, and 16 to <19 years. Rare visceral, severe craniocervical or spinal, and unclassified injuries were combined into a rare/other category because of small cell sizes. Differences in the distribution of primary injury categories across age groups were assessed using Pearson’s chi-square test with Monte Carlo estimation for sparse cell counts; Cramér’s V was calculated as effect size.

Factors associated with hospital admission were analyzed using multivariable logistic generalized estimating equations (GEEs) to account for repeated events within individual patients. The model included age, sex, fall versus non-fall mechanism, and primary injury category. Rare visceral, severe craniocervical or spinal, and unclassified injuries were combined into a rare/other category because of small cell sizes. Odds ratios with 95% confidence intervals are reported.

Sensitivity analyses of event-based comparisons were performed after restricting the dataset to the first chronological injury event per patient. Missing values were not imputed. All tests were two-sided, with *p* < 0.05 considered statistically significant. Statistical analyses were performed using Microsoft^®^ Excel for macOS (Version 16.42; Microsoft Corp., Redmond, WA, USA) and IBM^®^ SPSS^®^ Statistics (Version 27.0.0; IBM Corp., Armonk, NY, USA).

## 3. Results

### 3.1. Cohort Characteristics and Injury Mechanisms

A total of 650 horse-related injury events in 593 patients were included. Among the individual patients, 539 (90.9%) were female and 54 (9.1%) were male. At first presentation, the median age was 13.4 years (IQR, 10.8–16.0) in female patients and 10.7 years (IQR, 7.5–14.5) in male patients, corresponding to a median difference of 2.7 years. Considering all injury events, the respective median ages were 13.7 years (IQR, 11.0–16.1) and 10.9 years (IQR, 7.5–15.2), resulting in a difference of 2.8 years (Table 1).

At first presentation, the largest age group was 6 to <13 years (245/593, 41.3%), followed by 13 to <16 years (170/593, 28.7%) and 16 to <19 years (151/593, 25.5%); only 27 patients (4.6%) were younger than 6 years (Figure 1).

Notably, 45 patients sustained more than one separate horse-related injury event during the study period, accounting for 102 of the 650 events (15.7%; Table 2).

Falls from horses were the predominant injury mechanism, accounting for 454 of 650 events (69.8%), followed by kicks (*n* = 99, 15.2%), other horse-related mechanisms (*n* = 67, 10.3%), and bites (*n* = 30, 4.6%). The distribution of injury mechanisms was broadly comparable between sexes, with falls accounting for 69.9% of events in female and 69.6% in male patients. Kicks occurred at similarly comparable proportions (15.3% vs. 14.3%), whereas bites were proportionally more frequent among male patients (8.9% vs. 4.2%), although absolute numbers were small (Figure 2).

Information on protective equipment was available for only 22 of the 650 injury events (3.4%). Helmet use was documented in 19 events and body-protector use in three, whereas protective-equipment status was unknown for the remaining 628 events (96.6%).

### 3.2. Injury Patterns

Contusions were the most frequent primary injury category, accounting for 250 of 650 events (38.5%), followed by fractures in 159 events (24.5%), head injuries in 108 (16.6%), joint or ligamentous injuries in 73 (11.2%), and wounds in 49 (7.5%). Visceral injuries were recorded in four events (0.6%), and severe craniocervical or spinal injuries in three (0.5%). Four events (0.6%) could not be assigned to a sufficiently specific structural injury category (Figure 3).

#### 3.2.1. Association Between Injury Mechanism and Injury Pattern

Injury mechanism was significantly associated with primary injury pattern (χ^2^(15) = 358.5, *p* < 0.001; Cramer’s V = 0.43). Falls from horses were disproportionately associated with head injuries and fractures, kicks with contusions, wounds were predominantly caused by bites, and other horse-related mechanisms were associated with joint or ligamentous injuries. This association remained stable in the sensitivity analysis restricted to the first injury event of each patient (χ^2^(15) = 309.4, *p* < 0.001; Cramer’s V = 0.42).

#### 3.2.2. Contusions

Contusions were classified as the primary injury in 250 events (38.5%). Of these, 229 occurred in female and 21 in male patients, with a mean age of 13.4 ± 3.3 years. Falls from horses accounted for 161 events (64.4%), followed by kicks in 59 (23.6%), other horse-related mechanisms in 26 (10.4%), and bites in four (1.6%).

The lower extremity was affected in 101 events (40.4%), most commonly involving the foot (*n* = 39), thigh region (*n* = 23), knee (*n* = 12), toes (*n* = 10), and lower leg (*n* = 10). Upper-extremity contusions occurred in 61 events (24.4%), predominantly involving the shoulder (*n* = 16), forearm (*n* = 12), fingers (*n* = 11), and elbow (*n* = 9). Spinal contusions were documented in 36 events (14.4%), including the lumbar spine in 15, thoracic spine in nine, coccygeal region in eight, and cervical spine in four. Further locations included the thorax in 19 events (7.6%), abdomen in 13 (5.2%), neck in 11 (4.4%), and pelvis in nine (3.6%).

#### 3.2.3. Fractures

Fractures were classified as the primary injury in 159 events (24.5%). Of these, 139 occurred in female and 20 in male patients, with a mean age of 11.7 ± 3.6 years. Falls from horses accounted for 122 fractures (76.7%), kicks for 26 (16.4%), and other horse-related mechanisms for 11 (6.9%). No primary fracture resulted from a horse bite.

The fracture group comprised 98 conventional fractures (61.6%), 24 epiphysiolyses (15.1%), 15 buckle fractures (9.4%), eight bony avulsion fractures (5.0%), seven open fractures (4.4%), six fissures (3.8%), and one fracture-dislocation (0.6%).

The upper extremity was the most frequently affected anatomical region, accounting for 110 fractures (69.2%), followed by the lower extremity in 23 events (14.5%) and the craniofacial region in 16 (10.1%). Six fractures involved the spine (3.8%), while thoracic and pelvic fractures were documented in two events each (1.3%). Detailed fracture locations are presented in Table 3.

#### 3.2.4. Head Injuries and Traumatic Brain Injuries

Head injuries were classified as the primary injury in 108 events (16.6%). Of these, 102 occurred in female and six in male patients, with a mean age of 13.3 ± 3.5 years. Falls from horses accounted for 103 events (95.4%), followed by other horse-related mechanisms in three (2.8%) and kicks in two (1.9%). No primary head injury resulted from a bite.

Within this broad anatomical category, 59 events (54.6%) represented isolated head or craniofacial contusions without documented concussion or structural intracranial injury and were therefore considered non-TBI head injuries. The remaining 49 events (45.4%) fulfilled the study definition of traumatic brain injury (TBI), comprising 45 concussions (41.7% of all head injuries) and four intracranial hemorrhages (3.7%). Thus, TBIs accounted for 7.5% of all 650 horse-related injury events.

#### 3.2.5. Joint and Ligamentous Injuries

Joint or ligamentous injuries were classified as the primary injury in 73 events (11.2%). Of these, 70 occurred in female and three in male patients, with a mean age of 14.7 ± 2.4 years. Falls from horses accounted for 50 events (68.5%), other horse-related mechanisms for 19 (26.0%), and kicks for four (5.5%).

The group comprised 46 sprains or strains (63.0%), 17 ligament injuries (23.3%), four meniscal injuries (5.5%), four dislocations (5.5%), one cartilage injury (1.4%), and one muscle injury (1.4%).

The lower extremity was affected in 40 events (54.8%), most commonly involving the ankle (*n* = 22) and knee (*n* = 12), followed by the foot (*n* = 3), patellofemoral joint (*n* = 2), and lower leg (*n* = 1). Spinal or paraspinal injuries were documented in 18 events (24.7%), including 17 injuries of the cervical spine and one of the lumbar spine. The upper extremity was affected in 15 events (20.5%), predominantly involving the fingers (*n* = 9), shoulder (*n* = 2), and elbow (*n* = 2).

#### 3.2.6. Wounds

Wounds were classified as the primary injury in 49 events (7.5%). Of these, 44 occurred in female and five in male patients, with a mean age of 10.0 ± 5.1 years. Bites accounted for 26 events (53.1%), falls from horses for 13 (26.5%), kicks for six (12.2%), and other horse-related mechanisms for four (8.2%).

The group comprised 23 bite wounds (46.9%), 12 lacerations (24.5%), ten abrasions (20.4%), three soft-tissue defects (6.1%), and one partial amputation (2.0%).

The upper extremity was involved in 26 events (53.1%), predominantly affecting the fingers (*n* = 16), forearm (*n* = 7), and hand (*n* = 2). Thirteen wounds involved the head or face (26.5%), including ten facial and three scalp wounds. Six wounds affected the lower extremity (12.2%), while two thoracic and two pelvic wounds were recorded.

#### 3.2.7. Visceral, Craniocervical, and Spinal Injuries

Visceral injuries were classified as the primary injury in four events (0.6%), comprising three splenic ruptures and one liver rupture. Three occurred after falls from horses and one after a kick.

Severe craniocervical or spinal injuries were recorded in three events (0.5%). Two patients sustained fatal atlanto-occipital dislocations following falls from horses, while one sustained a non-fatal spinal cord injury during another horse-related mechanism. Vertebral fractures were included separately within the fracture category.

#### 3.2.8. Unclassified Injuries

Four events (0.6%) could not be assigned to a sufficiently specific structural injury category because of incomplete documentation. These comprised three presentations with pain without a clearly specified structural injury and one other unclear condition. They were retained as a separate category to avoid assigning an unsupported diagnosis.

#### 3.2.9. Age Distribution and Age-Related Differences in Injury Patterns

Age differed significantly across the five major primary injury categories—contusions, fractures, head injuries, joint or ligamentous injuries, and wounds (Kruskal–Wallis test, *p* < 0.001). Patients with wounds and fractures were younger, with median ages of 10 years (IQR, 7–15) and 12 years (IQR, 9–14), respectively, whereas joint or ligamentous injuries occurred predominantly in older patients (median, 15 years; IQR, 13–17). Holm-adjusted pairwise comparisons confirmed significant age differences between these groups and several of the remaining injury categories. The overall finding remained unchanged when the analysis was restricted to the first injury event of each patient (*p* < 0.001).

In the additional age-stratified analysis, the distribution of primary injury categories differed significantly across age groups (χ^2^(15) = 101.01, *p* < 0.001; Cramér’s V = 0.23). Wounds were proportionally most frequent among children aged <6 years (40.7%). Fractures accounted for 34.2% of injuries among children aged 6 to <13 years and decreased progressively in the older age groups. In contrast, joint or ligamentous injuries were substantially more frequent among adolescents aged 13 to <16 years (17.0%) and 16 to <19 years (16.6%). The association remained materially unchanged when the analysis was restricted to the first injury event per patient (χ^2^(15) = 91.05, *p* < 0.001; Cramér’s V = 0.23) (Table 4).

### 3.3. Hospitalization, Treatment, and Documented Outcomes

#### 3.3.1. Hospitalization

Overall, 113 of 650 injury events (17.4%) required inpatient treatment, whereas 537 (82.6%) were managed on an outpatient basis. Length of hospital stay was available for 112 admitted events and had a median duration of 3 days (IQR, 2–4; mean, 4.8 days; range, 1–32). Fractures and head injuries accounted for 85 of the 113 hospital admissions (75.2%).

In the multivariable GEE model accounting for repeated injury events within individual patients, fractures (adjusted odds ratio [aOR], 5.43; 95% CI, 2.86–10.31), head injuries (aOR, 8.85; 95% CI, 4.54–17.28), and rare or other injuries (aOR, 34.68; 95% CI, 9.97–120.61) were independently associated with hospital admission compared with contusions. Falls from horses were also associated with higher odds of inpatient treatment compared with non-fall mechanisms (aOR, 2.00; 95% CI, 1.01–3.96). Age and sex were not independently associated with hospital admission (Table 5). In the sensitivity analysis restricted to the first injury event of each patient, the associations between injury category and hospital admission remained materially unchanged.

#### 3.3.2. Treatment and Clinical Course

Most injury events were managed non-operatively (*n* = 616, 94.8%), while operative treatment was required in 34 injury events (5.2%). Fracture-related procedures accounted for 26 of these 34 surgically treated events (76.5%). At least one subsequent operation was documented in 20 events, corresponding to 3.1% of the entire cohort and 58.8% of surgically treated events. Across these 20 events, a total of 34 subsequent procedures were recorded. These included planned implant removal as well as staged soft-tissue, reconstructive, and revision procedures.

#### 3.3.3. Follow-Up and Documented Outcomes

Follow-up beyond the initial presentation was documented in 236 events (36.3%), with a median duration of 17 days (IQR, 4–29; range, 1–564). In 401 events (61.7%), treatment was completed during a single outpatient visit and no further follow-up was scheduled. In 13 events (2.0%), follow-up information was unavailable because further treatment was provided by another department.

Among the 234 non-fatal events with documented follow-up, 195 (83.3%) were free of complaints at the final assessment, whereas 39 (16.7%) had persistent symptoms or clinical impairment. Two patients died following fatal atlanto-occipital dislocations (0.3%). Detailed data on hospitalization, treatment, follow-up, and documented outcomes are presented in Table 6.

To assess the potential impact of incomplete follow-up, a scenario analysis was performed using extreme assumptions for the 414 non-fatal events without documented outcome assessment. Assuming that all 401 events managed during a single outpatient visit remained free of complaints, while all 13 events with unavailable follow-up had persistent symptoms, 596 of 648 non-fatal events (92.0%) would be classified as complaint-free. Conversely, if all 414 events without documented outcome assessment were assumed to have persistent symptoms, 453 of 648 events (69.9%) would be classified as having persistent symptoms. These scenarios illustrate the uncertainty introduced by incomplete follow-up and support interpreting the documented outcome proportions as conditional on follow-up availability.

### 3.4. Fatal Cranio-Cervical Injuries

#### 3.4.1. Case 1

A 4-year-old girl fell during guided pony riding and was dragged for approximately 300 m after her foot became caught in the stirrup. She became unresponsive at the scene, and cardiopulmonary resuscitation was initiated. Return of spontaneous circulation was achieved approximately 10 min after arrival of the emergency medical team. The patient was intubated, mechanically ventilated, and transferred by helicopter to the trauma resuscitation unit.

On admission, the right pupil was fixed and dilated. External signs of trauma were limited to left-sided facial abrasions, periorbital hematoma, and epistaxis. Computed tomography demonstrated marked craniocervical distraction with displacement of the skull relative to C1 by up to 2 cm and atlanto-occipital joint dislocation (Figure 4). The posterior arch of C1 was displaced, and marked swelling of the spinal cord at C1–C2 was accompanied by subdural and epidural hemorrhage. Additional intracranial findings included mild cerebral edema and falcine and bilateral frontoparietal subdural hemorrhage; a nasal bone fracture was also present.

Intensive care treatment was initiated. Brain death was subsequently confirmed, and organ procurement was performed two days after the injury.

#### 3.4.2. Case 2

A 12-year-old girl fell from a horse while riding independently and became caught in the stirrup, resulting in her being dragged for an unknown distance. She initially remained conscious and was able to stand after the accident. Shortly thereafter, however, she developed generalized convulsions, collapsed, and became unresponsive. Bystander cardiopulmonary resuscitation was initiated and continued until arrival of the emergency medical team. The patient was intubated and mechanically ventilated, and cardiovascular stability was subsequently achieved. Bilaterally fixed and dilated pupils were documented at the scene.

Her medical history was notable for hydrocephalus treated with ventriculoperitoneal shunt placement three years previously. On admission, no major external signs of trauma were apparent. According to the initial trauma CT report, imaging demonstrated severe diffuse cerebral swelling with effacement of the basal cisterns and an approximately 10-mm leftward midline shift, consistent with cerebral herniation. No intracranial hemorrhage was reported. An injury of the craniocervical junction was not described in the initial CT report. Subsequent conventional cervical spine radiography demonstrated an atlanto-occipital dislocation without an associated cervical spine fracture (Figure 5).

This case highlights an important diagnostic pitfall. The atlanto-occipital dislocation was not described in the initial trauma CT report, which primarily documented the intracranial findings, and was subsequently identified on lateral cervical spine radiography. This underscores the importance of dedicated assessment of the craniocervical junction in children after high-energy equestrian trauma, particularly when the mechanism involves entrapment and dragging despite limited external signs of injury.

Despite intensive care treatment, brain death was confirmed, and organ procurement was performed on the following day.

## 4. Discussion

The present study provides a broad characterization of pediatric horse-related trauma across a 22-year period and highlights three clinically relevant findings. First, the majority of injuries were managed on an outpatient and non-operative basis, demonstrating that horse-related trauma encompasses a considerably broader clinical spectrum than severe injuries alone. Second, injury mechanism was strongly associated with the resulting injury pattern, suggesting distinct mechanism-specific injury phenotypes. Third, the most frequent injuries were not necessarily those responsible for the greatest treatment burden: although contusions predominated overall, fractures and head injuries accounted for a disproportionate share of hospital admissions. Two fatal atlanto-occipital dislocations further illustrate the rare but potentially catastrophic end of this injury spectrum.

The marked predominance of female patients and the high proportion of falls observed in the present cohort are consistent with previous pediatric studies [5,6,9,13]. Many published series, however, have been derived from hospitalized patients, trauma registries, or selected injury groups and therefore naturally emphasize fractures, head injuries, operative treatment, and other severe outcomes [14,15,16]. In the present cohort, only 17.4% of injury events required hospital admission and 5.2% underwent operative treatment. The inclusion of both outpatient and hospitalized patients therefore provides a broader view of the clinical spectrum [9,13] encountered at a trauma center, ranging from frequent minor injuries to uncommon life-threatening events [3,17]. This distinction is important when interpreting the epidemiology of horse-related trauma, as cohorts restricted to admitted or severely injured patients [15,16] may not reflect the distribution of injuries seen in routine clinical practice.

A central finding of the present study was the strong association between injury mechanism and primary injury pattern. Previous pediatric studies have demonstrated clinically relevant differences between mounted and unmounted injuries [9,18]. The present findings extend this concept by differentiating specific mechanisms of trauma. Falls were primarily associated with fractures and head injuries, kicks with contusions, and bites with wounds. Rather than representing a single homogeneous clinical entity [15], horse-related trauma therefore comprises distinct injury profiles according to the circumstances of the event [9,18]. From a clinical perspective, this may be useful during the initial assessment of an injured child, as the reported mechanism can provide an early indication of the injuries that should be anticipated and actively excluded.

Age-related differences in injury pattern provide additional support for this heterogeneity. Fractures and wounds occurred in younger children, whereas joint or ligamentous injuries were predominantly observed in older patients. Previous pediatric studies have likewise demonstrated age-related differences in injury mechanism and anatomical location, with younger children also showing greater injury severity in selected cohorts [9,14,18]. These differences may reflect age-dependent patterns of exposure, riding experience, supervision, or interaction with horses. However, detailed information regarding these factors was not consistently available in the present dataset, and the underlying reasons for the observed age differences therefore remain speculative.

The distinction between injury frequency and clinical burden was particularly evident in the analysis of hospital admission. Contusions represented the most frequent injury category overall, but fractures and head injuries together accounted for the majority of inpatient treatment. After adjustment for age, sex, injury mechanism, and repeated injury events within individual patients, fractures were associated with more than fivefold and head injuries with almost ninefold higher odds of hospital admission compared with contusions. These findings support previous observations that fractures and head injuries are major contributors to morbidity in pediatric equestrian trauma [5,8,16,19], while further quantifying their independent contribution to inpatient treatment. The results also emphasize that injury frequency alone should not be interpreted as a measure of clinical relevance. Relatively less frequent injury categories may account for a disproportionate share of resource-intensive care.

The two fatal atlanto-occipital dislocations represent the most severe end of this injury spectrum. Atlanto-occipital dislocation is a rare and highly unstable craniocervical injury in children and is predominantly described following high-energy road-traffic trauma [11,12]. Published pediatric horse-related trauma series have not generally identified atlanto-occipital dislocation as a distinct injury entity [9,13]. Remarkably, both patients in the present cohort sustained their injuries following a similar complex mechanism involving a fall, entrapment in the stirrup, and subsequent dragging. Both subsequently developed profound neurological deterioration despite relatively limited external signs of trauma. In one patient, injury of the craniocervical junction was not described in the initial CT report and was subsequently demonstrated on cervical spine radiography. Although two cases do not permit inference regarding a specific risk attributable to stirrup entrapment or dragging, their similarity highlights the potential for devastating craniocervical injury following complex equestrian trauma and the importance of maintaining a high index of suspicion after high-energy mechanisms [11,12]. In such circumstances, dedicated evaluation of the craniocervical junction using sagittal and coronal CT reconstructions, including assessment of the condylar-C1 and basion-dens intervals, should be considered. Where ligamentous injury remains suspected, MRI may provide additional information.

Protective equipment remains an important component of injury prevention, particularly with regard to head trauma [6,20,21]. In the present cohort, helmet or body-protector use was documented in only 22 of 650 injury events (3.4%), while protective-equipment status was unknown for the remaining 96.6%. This substantial documentation gap represents a major limitation, as it precluded any meaningful assessment of a potential protective effect and may reflect under-documentation rather than true non-use. It also illustrates a limitation of retrospective injury surveillance based on routine clinical documentation. Future prospective equestrian injury registries should therefore systematically record helmet and body-protector use, riding versus handling status, level of supervision, riding experience, horse size, and detailed accident mechanisms, including stirrup entrapment and dragging, using a standardized template at initial presentation [13].

Several additional limitations should be considered. The retrospective single-center design depended on the completeness and accuracy of routine clinical documentation. In particular, keyword-based case identification may have missed eligible horse-related injury events if the mechanism was documented using terminology not captured by the predefined search terms, and incomplete case ascertainment therefore cannot be excluded. The long observation period may also encompass changes in imaging, documentation, treatment practices, and equestrian safety. Follow-up beyond the initial presentation was documented in only 236 events (36.3%), with a median duration of 17 days, limiting assessment of long-term outcomes and potentially underestimating longer-term morbidity. Exposure data were unavailable, preventing calculation of injury incidence or individual risk. Most analyses were performed at the injury-event level rather than the patient level; repeated events within individual patients were therefore accounted for using GEE models and sensitivity analyses restricted to the first chronological injury event per patient. Classification according to the clinically most relevant primary injury necessarily simplified events involving multiple injuries. Conversely, the large cohort, long observation period, inclusion of both outpatient and hospitalized patients, detailed clinical reclassification, consideration of repeated injury events, and multivariable analysis provide a comprehensive perspective on the spectrum and clinical relevance of pediatric horse-related trauma.

## 5. Conclusions

Pediatric horse-related trauma encompasses a broad clinical spectrum, with most injuries managed on an outpatient and non-operative basis. However, specific injury mechanisms are associated with distinct injury patterns, and the most frequent injuries are not necessarily those associated with the greatest treatment burden. Fractures and head injuries accounted for a disproportionate share of hospital admissions, while rare complex mechanisms may result in devastating craniocervical trauma. Recognition of mechanism-specific injury profiles may support targeted clinical assessment and help identify children at risk for more severe injury. Prospective standardized documentation of protective equipment, riding circumstances, and detailed accident mechanisms may further improve injury surveillance and prevention strategies.

## Figures and Tables

**Figure 1:**
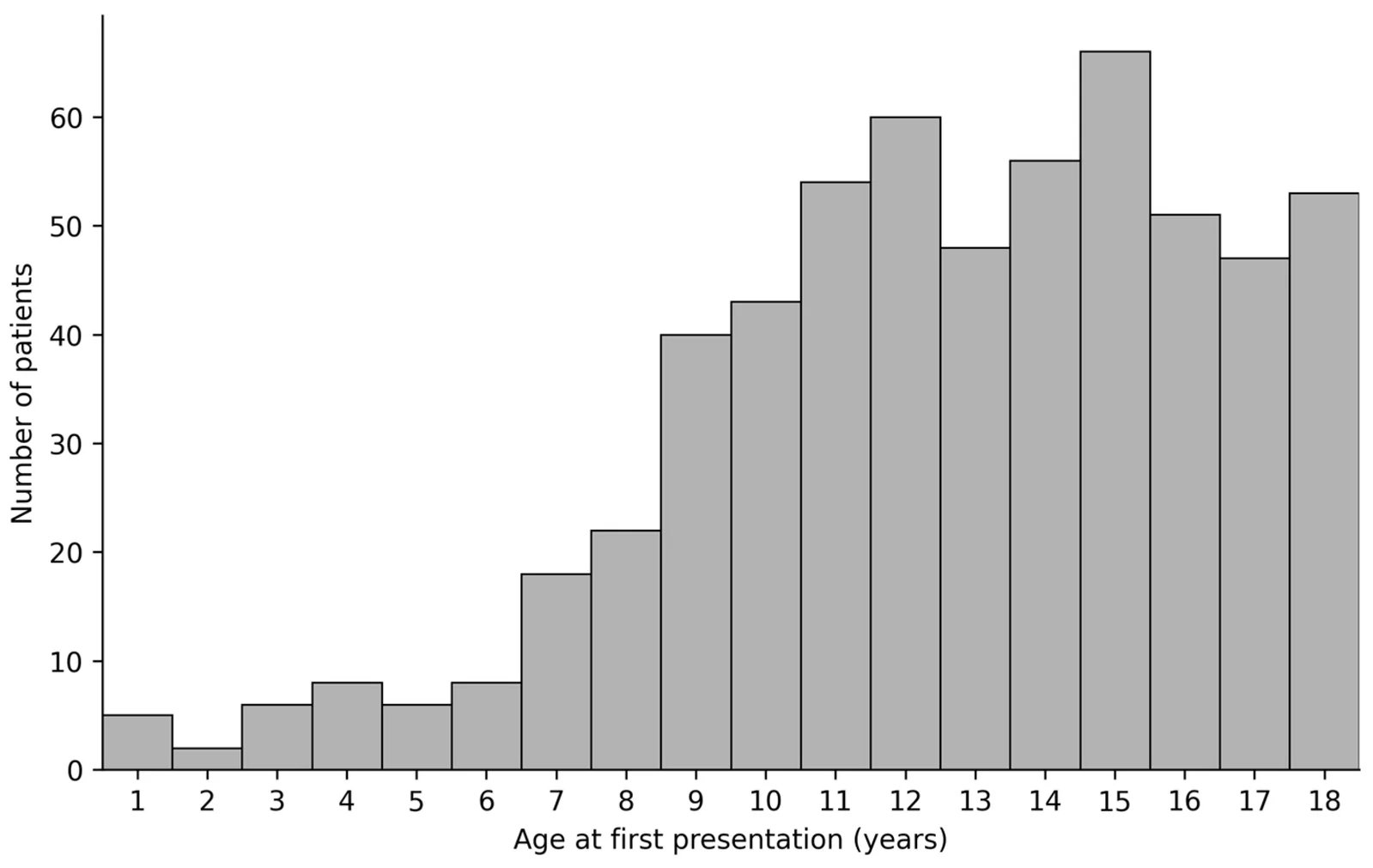
Age distribution at first presentation among 593 individual patients.

**Figure 2:**
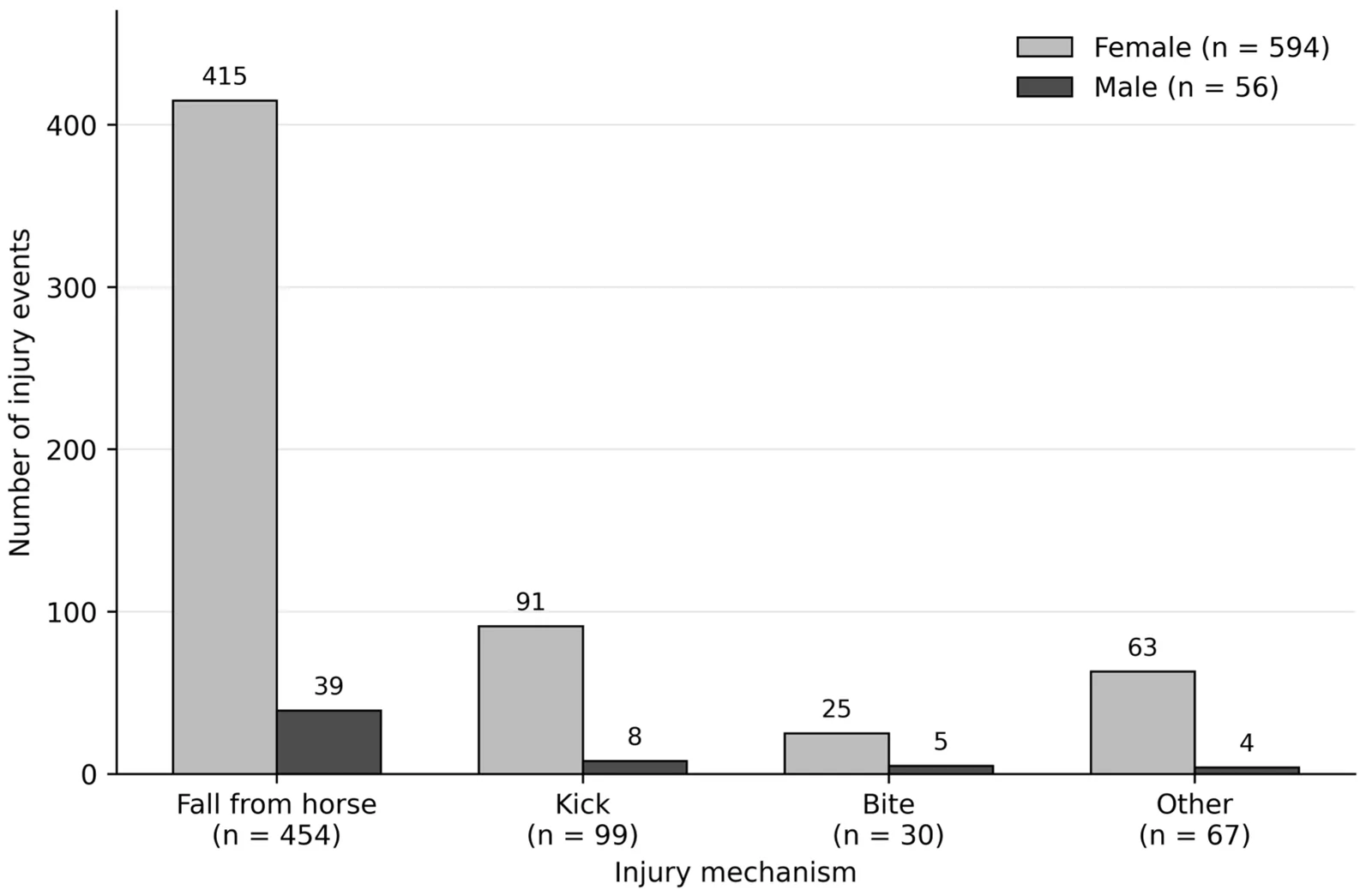
Distribution of horse-related injury mechanisms according to sex.

**Figure 3:**
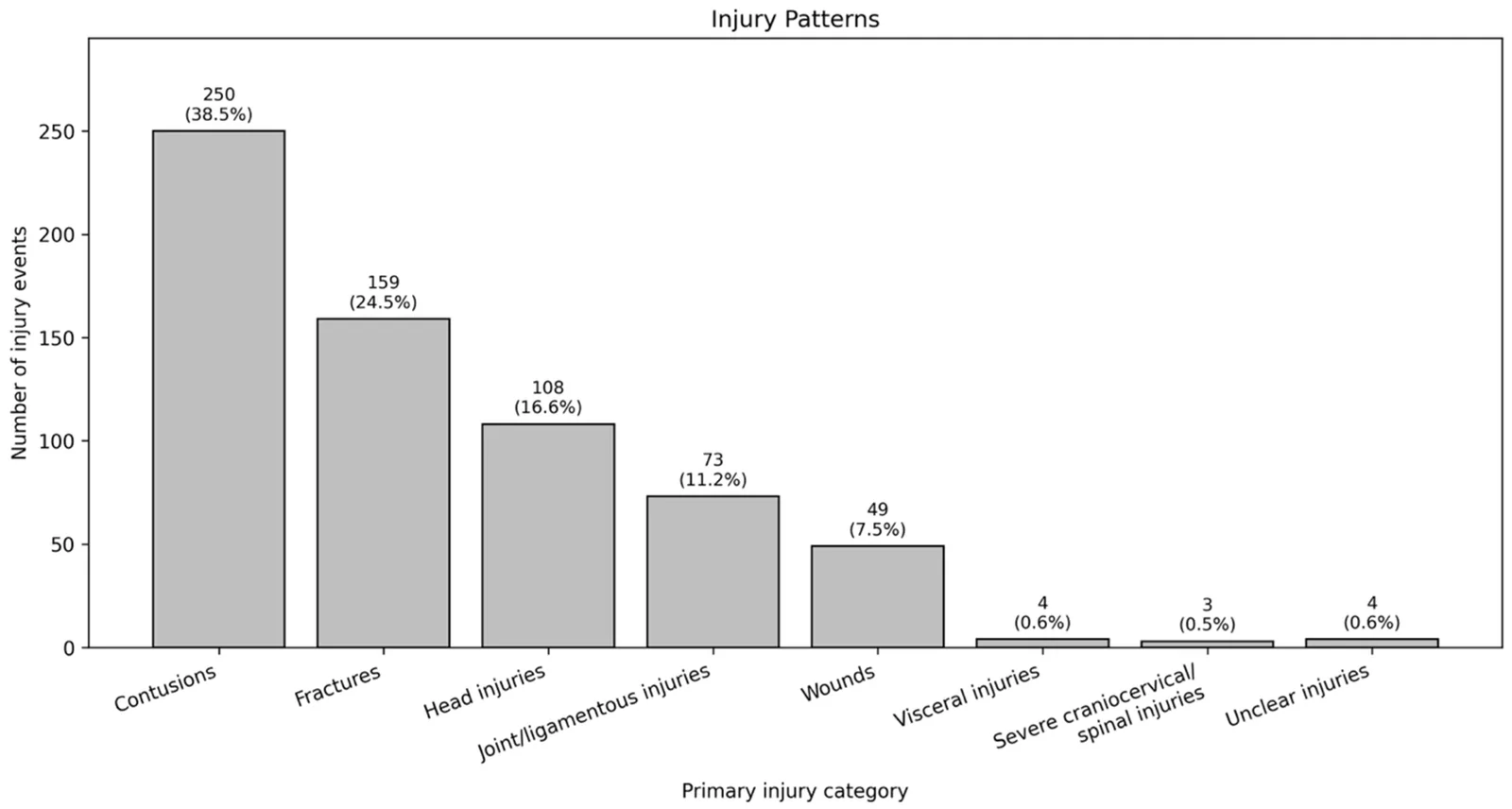
Distribution of primary injury categories among 650 horse-related injury events.

**Figure 4:**
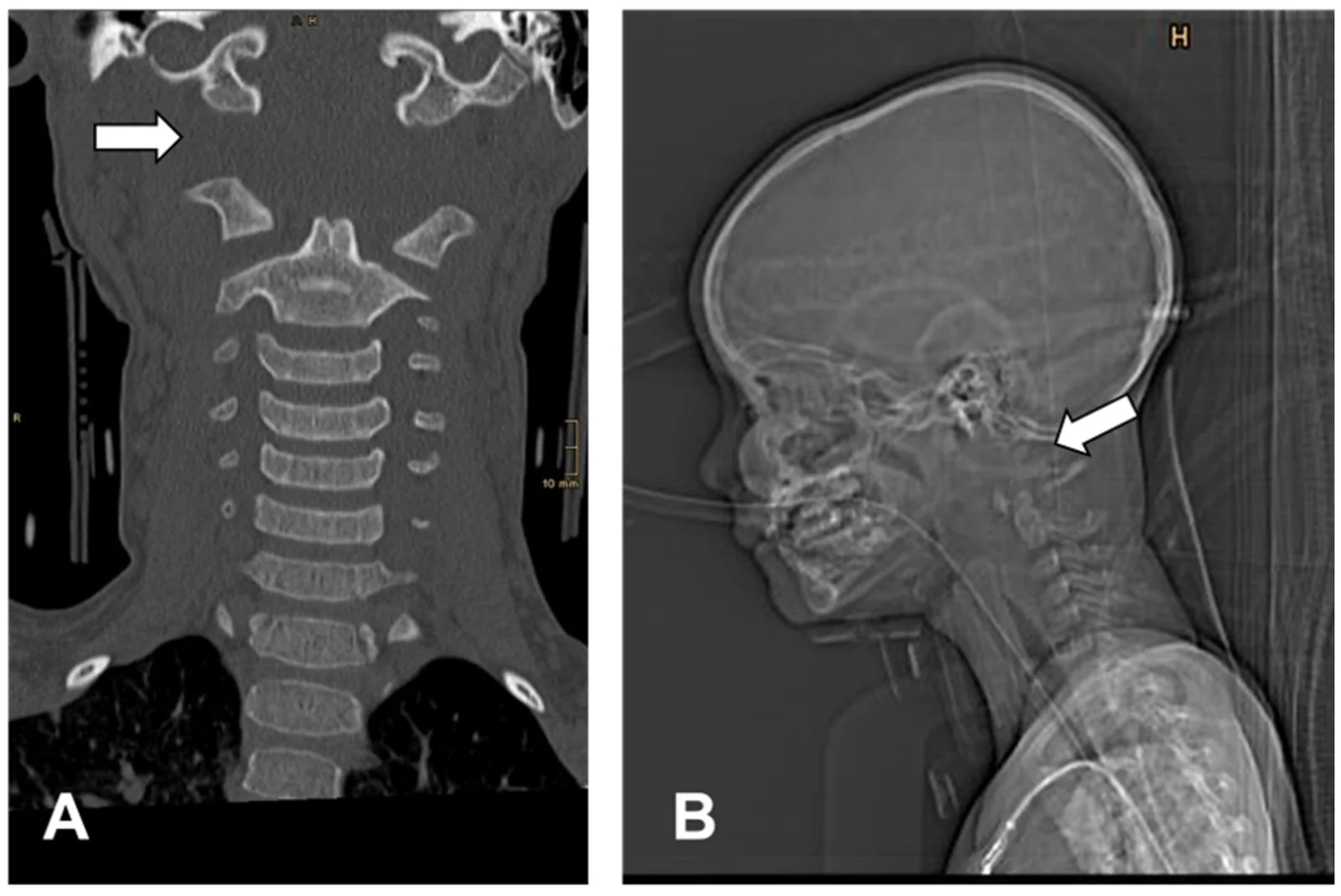
Computed tomography of Patient 1 demonstrating marked craniocervical distraction with atlanto-occipital dislocation on coronal CT reconstruction (**A**) and lateral CT topogram (**B**).

**Figure 5:**
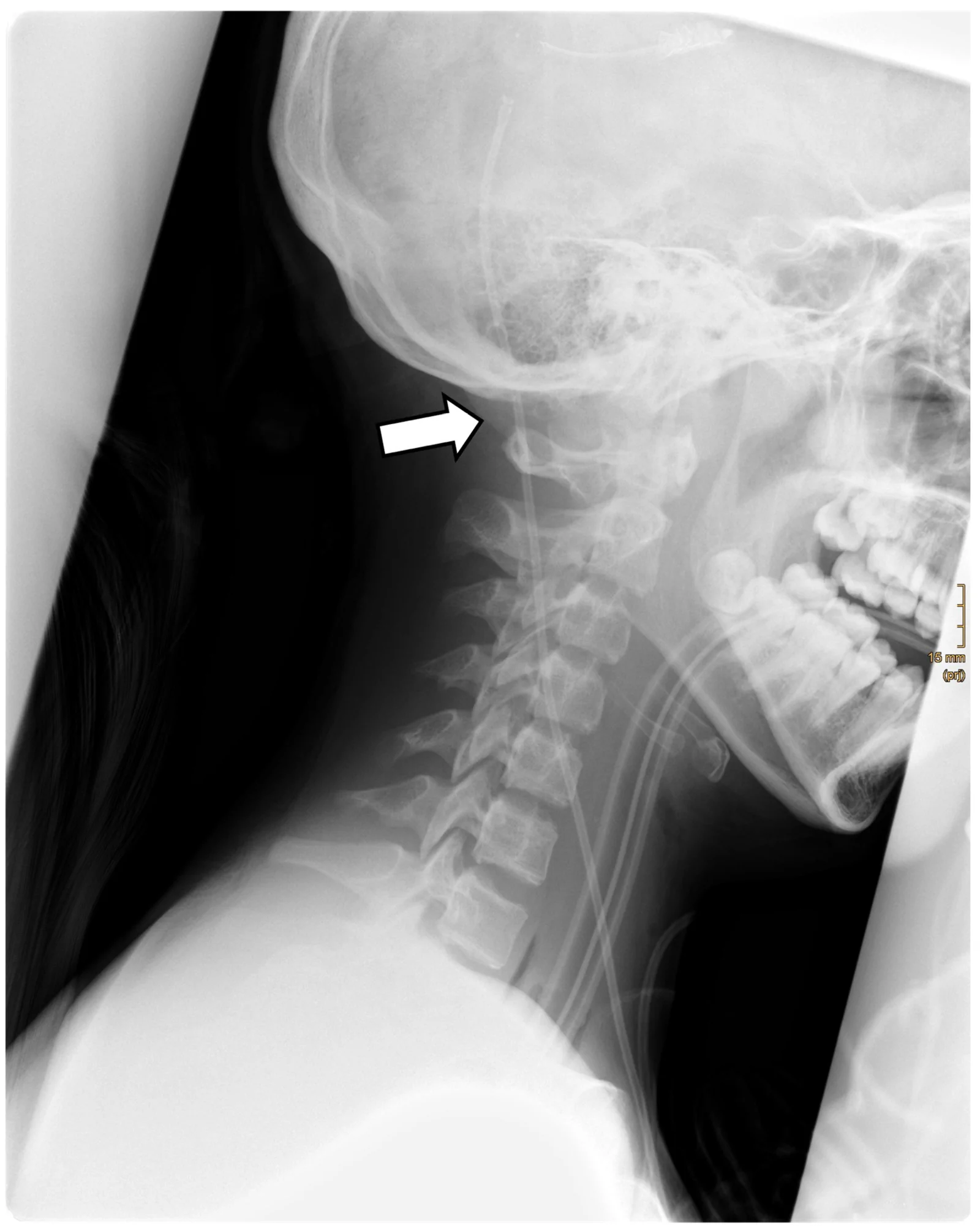
Lateral cervical spine radiograph of Patient 2 demonstrating atlanto-occipital dislocation without an associated cervical spine fracture. Sagittal CT reconstruction was not available for this patient.

**Table 1:** Age distribution at first presentation and across all injury events, stratified by sex.

Sex	*n*	M ± SD	Min–Max	Md (IQR)
At first presentation				
female	539	13.2 ± 3.7	1.0–18.9	13.4 (10.8; 16.0)
male	54	10.8 ± 4.9	1.5–18.9	10.7 (7.5; 14.5)
Total	593	13.0 ± 3.8	1.0–18.9	13.3 (10.5; 15.9)
All injury events				
female	594	13.4 ± 3.7	1.0–18.9	13.7 (11.0; 16.1)
male	56	11.0 ± 4.9	1.5–18.9	10.9 (7.5; 15.2)
Overall	650	13.2 ± 3.8	1.0–18.9	13.5 (10.7; 16.1)

Footnote: Md, median; IQR, interquartile range; M, mean; SD, standard deviation; *n*, number of patients or injury events, as applicable.

**Table 2:** Distribution of horse-related injury events per patient during the study period.

Injury Events per Patient	Patients (*n*)	Injury Events (*n*)	Proportion of all Injury Events (%)
1	548	548	84.3%
2	36	72	11.1%
3	7	21	3.2%
4	1	4	0.6%
5	1	5	0.8%
Total	593	650	100%

**Table 3:** Anatomical distribution of fractures.

Anatomical Region	Fracture Location	*n*	% of All Fractures
Upper extremity	Wrist/distal radius	31	19.5
	Forearm	20	12.6
	Elbow/supracondylar region	19	11.9
	Humerus	15	9.4
	Hand/fingers	13	8.2
	Clavicle/scapula	12	7.5
Lower extremity	Foot/Toes	13	8.2
	Ankle	6	3.8
	Femur	2	1.3
	Tibia/fibula	2	1.3
Craniofacial region	Facial skeleton	10	6.3
	Nasal bone	5	3.1
	Skull	1	0.6
Spine	Lumbar spine	5	3.1
	Coccygeal region	1	0.6
Other regions	Thorax	2	1.3
	Pelvis	2	1.3
Total		159	100

**Table 4:** Distribution of primary injury categories according to age group.

Injury Category	<6 Years *n* (%)	6–<13 Years *n* (%)	13–<16 Years *n* (%)	16–<19 Years *n* (%)	Total *n*
Contusions	6 (22.2)	89 (34.2)	79 (42.0)	76 (43.4)	250
Fractures	7 (25.9)	89 (34.2)	37 (19.7)	26 (14.9)	159
Head Injuries	1 (3.7)	45 (17.3)	27 (14.4)	35 (20.0)	108
Joint/ligamentous injuries	0 (0.0)	12 (4.6)	32 (17.0)	29 (16.6)	73
Wounds	11 (40.7)	21 (8.1)	11 (5.9)	6 (3.4)	49
Rare/other injuries	2 (7.4)	4 (1.5)	2 (1.1)	3 (1.7)	11
Total	27	260	188	175	650

Footnote: Values are presented as *n* (% within age group). Rare/other injuries comprise visceral injuries, severe craniocervical or spinal injuries, and unclassified injuries, which were combined because of small cell sizes.

**Table 5:** Factors associated with hospital admission.

Variable	Hospitalized *n*/N (%)	Crude OR (95% CI)	Adjusted OR (95% CI)	*p* Value
Age, per year	-	0.99 (0.94–1.05)	1.03 (0.97–1.09)	0.353
Sex				
Female	101/594 (17.0)	Reference		-
Male	12/56 (21.4)	1.33 (0.67–2.62)	1.30 (0.61–2.78)	0.491
Mechanism				
Non-fall mechanism	17/196 (8.7)	Reference		-
Fall from horse	96/454 (21.1)	2.81 (1.63–4.86)	2.00 (1.01–3.96)	0.046
Injury category				
Contusion	15/250 (6.0)	Reference		-
Fracture	42/159 (26.4)	5.54 (2.96–10.34)	5.43 (2.86–10.31)	<0.001
Head injury	43/108 (39.8)	10.33 (5.41–19.72)	8.85 (4.54–17.28)	<0.001
Joint/ligamentous injury	2/73 (2.7)	0.43 (0.10–1.87)	0.41 (0.09–1.80)	0.237
Wound	4/49 (8.2)	1.38 (0.44–4.36)	1.99 (0.59–6.68)	0.268
Rare/other injury	7/11 (63.6)	27.19 (7.22–102.40)	34.68 (9.97–120.61)	<0.001

Footnote: OR, odds ratio; CI, confidence interval; aOR, adjusted odds ratio. Reference categories were female sex, non-fall mechanism, and contusion. Rare/other injuries comprise visceral injuries, severe craniocervical or spinal injuries, and unclassified injuries.

**Table 6:** Clinical course and outcome.

Clinical Parameter	*n*/N (%)	Age, Years	Female, *n* (%)	Male, *n* (%)
Management setting				
Outpatient	537/650 (82.6)	12.9 ± 3.8	493 (91.8)	44 (8.2)
Inpatient	113/650 (17.4)	12.7 ± 3.5	101 (89.4)	12 (10.6)
Length of stay, days	Median 3 (IQR, 2–4); range, 1–32			
Treatment				
Non-operative	616/650 (94.8)	12.9 ± 3.7	564 (91.6)	52 (8.4)
Operative	34/650 (5.2)	12.2 ± 3.4	30 (88.2)	4 (11.8)
≥1 subsequent procedure	20/34 (58.8)	11.5 ± 3.5	16 (80.0)	4 (20.0)
Follow-up				
Follow-up interval > 0 days	236/650 (36.3)	12.4 ± 3.8	208 (88.1)	28 (11.9)
Follow-up duration, days	Median 17 (IQR, 4–29); range, 1–564			
Follow-up = 0 days	401/650 (61.7)	13.1 ± 3.7	373 (93.0)	28 (7.0)
Follow-up unavailable	13/650 (2.0)	13.5 ± 2.9	13 (100.0)	-
Documented outcomes				
No complaints	195/234 (83.3)	12.3 ± 3.7	174 (89.2)	21 (10.8)
Persistent symptoms	39/234 (16.7)	13.0 ± 3.8	32 (82.1)	7 (17.9)
Death	2/650 (0.3)	4 and 12	2 (100.0)	-

Footnote: Age is presented as mean ± SD; individual ages are shown for fatal cases.

## Data Availability

The datasets generated and/or analyzed in the current study are not publicly available due to data privacy but are available from the corresponding author on reasonable request.

## Supplementary Materials

The following supporting information can be downloaded at: https://www.mdpi.com/article/10.3390/pediatric18050126/s1, Table S1: Breakdown of injury events classified as “other horse-related mechanisms” (*n* = 67).

## References

[1] Neville E.K., Hicks H., Neville C.C. (2024). Epidemiology of horse trauma: A literature review. Eur. J. Trauma Emerg. Surg..

[2] Kruger L., Hohberg M., Lehmann W., Dresing K. (2018). Assessing the risk for major injuries in equestrian sports. BMJ Open Sport Exerc. Med..

[3] Cuenca A.G., Wiggins A., Chen M.K., Kays D.W., Islam S., Beierle E.A. (2009). Equestrian injuries in children. J. Pediatr. Surg..

[4] Gates J.K., Lin C.Y. (2020). Head and Spinal Injuries in Equestrian Sports: Update on Epidemiology, Clinical Outcomes, and Injury Prevention. Curr. Sports Med. Rep..

[5] Holland A.J.A., Roy G.T., Goh V., Ross F.I., Keneally J.P., Cass D.T. (2001). Horse-related injuries in children. Med. J. Aust..

[6] Theodore J.E., Theodore S.G., Stockton K.A., Kimble R.M. (2017). Paediatric horse-related trauma. J. Paediatr. Child Health.

[7] Naef S., Steiger C.N., Tabard-Fougère A., Dayer R., Ceroni D. (2022). Epidemiological Study of Equestrian Trauma in Geneva. J. Pediatr. Orthop..

[8] Ghosh A., DiScala C., Drew C., Lessin M., Feins N. (2000). Horse-related injuries in pediatric patients. J. Pediatr. Surg..

[9] Wolyncewicz G.E.L., Palmer C.S., Jowett H.E., Hutson J.M., King S.K., Teague W.J. (2018). Horse-related injuries in children—Unmounted injuries are more severe: A retrospective review. Injury.

[10] Craven J.A. (2008). Paediatric and adolescent horse-related injuries: Does the mechanism of injury justify a trauma response?. Emerg. Med. Australas..

[11] Beez T., Brown J. (2017). Traumatic atlanto-occipital dislocation in children-a case-based update on clinical characteristics, management and outcome. Childs Nerv. Syst..

[12] Astur N., Sawyer J.R., Klimo P., Kelly D.M., Muhlbauer M., Warner W.C. (2014). Traumatic atlanto-occipital dislocation in children. J. Am. Acad. Orthop. Surg..

[13] Boije Af Gennas K., Jungmalm J. (2025). Youth horse-related injuries (2014–2024): A scoping review of epidemiological and disciplinary insights. BMJ Open Sport Exerc. Med..

[14] Loder R.T., Walker A.L., Blakemore L.C. (2024). Injury Patterns and Associated Demographic Characteristics in Children with a Fracture from Equines: A US National Based Study. Children.

[15] Nguyen H.S., Lew S. (2016). Equestrian-Related Traumatic Brain Injury in the Pediatric Population. Pediatr. Neurosurg..

[16] Gross I., Hadar A., Bala M., Hashavya S. (2019). The Epidemiology, Injury Patterns and Outcomes of Horse-Related Injuries in Israeli Children. Isr. Med. Assoc. J..

[17] Altgarde J., Redéen S., Hilding N., Drott P. (2014). Horse-related trauma in children and adults during a two year period. Scand. J. Trauma Resusc. Emerg. Med..

[18] Kiss K., Swatek P., Lénárt I., Mayr J., Schmidt B., Pintér A., Höllwarth M.E. (2008). Analysis of horse-related injuries in children. Pediatr. Surg. Int..

[19] Jagodzinski T., DeMuri G.P. (2005). Horse-related injuries in children: A review. Wis. Med. J..

[20] Bond G.R., Christoph R.A., Rodgers B.M. (1995). Pediatric equestrian injuries: Assessing the impact of helmet use. Pediatrics.

[21] Short S.S., Fenton S.J., Scaife E.R., Bucher B.T. (2018). Helmet under-utilization by children during equestrian events is associated with increased traumatic brain injury. J. Pediatr. Surg..

