# Supplementary material for: From Contusions to Fatal Atlanto-Occipital Dislocations: The Clinical Spectrum of Pediatric Horse-Related Trauma over 22 Years
Source: Pediatr Rep. 2026 Sep 25;18(5):126. doi: 10.3390/pediatric18050126 (PMC13708669; doi:10.3390/pediatric18050126)
Supplement: Supplementary file 1 [file pediatrrep-18-00126-s001.zip › pediatrrep-4571702-supplementary.pdf]

Table S1. Breakdown of injury events classified as "other horse-related mechanisms" (n = 67).

Other horse-related mechanism	n	%
Riding-related injury without a fall	20	29.8
Direct impact, collision or compression	16	23.9
Injury during mountin, dismounting or jumping from the horse	13	19.4
Entrapment or dragging	7	10.4
Injury while handling, leading, lunging, saddling or holding the horse	6	9.0
Fall during handling	3	4.5
Unspecified or unclear mechanism	2	3.0
**Total**	**67**	**100.0**

Footnote: "Other horse-related mechanisms" comprised injury events that could not be classified as a fall from the horse, kick, or bite. Categories were derived from the documented accident descriptions.
